# Multidimensional pain phenotypes after Traumatic Brain Injury

**DOI:** 10.3389/fpain.2022.947562

**Published:** 2022-08-19

**Authors:** Linda E. Robayo, Varan Govind, Roberta Vastano, Elizabeth R. Felix, Loriann Fleming, Nicholas P. Cherup, Eva Widerström-Noga

**Affiliations:** ^1^Neuroscience Graduate Program, University of Miami Miller School of Medicine, Miami, FL, United States; ^2^Christine E. Lynn Rehabilitation Center, Miami Project to Cure Paralysis at UHealth/Jackson Memorial, Miami, FL, United States; ^3^Department of Radiology, Miller School of Medicine, University of Miami, Miami, FL, United States; ^4^Department of Neurological Surgery, University of Miami Miller School of Medicine, Miami, FL, United States; ^5^Department of Physical Medicine and Rehabilitation, University of Miami Miller School of Medicine, Miami, FL, United States

**Keywords:** chronic pain, Traumatic Brain Injury, pain phenotypes, psychological distress, neuropathic pain, quantitative somatosensory testing

## Abstract

More than 50% of individuals develop chronic pain following traumatic brain injury (TBI). Research suggests that a significant portion of post-TBI chronic pain conditions is neuropathic in nature, yet the relationship between neuropathic pain, psychological distress, and somatosensory function following TBI is not fully understood. This study evaluated neuropathic pain symptoms, psychological and somatosensory function, and psychosocial factors in individuals with TBI (TBI, *N* = 38). A two-step cluster analysis was used to identify phenotypes based on the Neuropathic Pain Symptom Inventory and Beck's Anxiety Inventory scores. Phenotypes were then compared on pain characteristics, psychological and somatosensory function, and psychosocial factors. Our analyses resulted in two different neuropathic pain phenotypes: (1) Moderate neuropathic pain severity and anxiety scores (MNP-AS, *N* = 11); and (2) mild or no neuropathic pain symptoms and anxiety scores (LNP-AS, *N* = 27). Furthermore, the MNP-AS group exhibited greater depression, PTSD, pain severity, and affective distress scores than the LNP-AS group. In addition, thermal somatosensory function (difference between thermal pain and perception thresholds) was significantly lower in the MNP-AS compared to the LNP-AS group. Our findings suggest that neuropathic pain symptoms are relatively common after TBI and are not only associated with greater psychosocial distress but also with abnormal function of central pain processing pathways.

## Introduction

Traumatic brain injury (TBI) affects millions of people per year in the United States (1). In addition to cognitive impairments (2, 3), individuals may also develop chronic pain across multiple body regions (4–6), which can be debilitating and contribute to long-term opioid use during recovery (7, 8). Neuropathic pain symptoms have been reported by those who have experienced a TBI (4, 9, 10), suggesting the presence of central neuropathic pain, which is defined as “pain caused by a lesion or disease of the central somatosensory nervous system” (11). Similarly, the recent 11^th^ revision of the International Classification of Diseases and Related Health Problems (ICD-11) included “central neuropathic pain associated with brain injury” under chronic pain conditions (12, 13). While neuropathic pain (14, 15) is well documented in individuals with spinal cord injury (SCI) (16), stroke (17), and multiple sclerosis (18), much less is known about neuropathic pain associated with TBI, including its prevalence (4, 5, 9, 10, 19).

Post-traumatic stress disorder (PTSD), depression, anxiety, fatigue, and sleep disorders are common comorbidities associated with TBI (9, 20–26) and can be etiologically linked to the development of chronic pain (20, 27, 28). The simultaneous assessment of neuropathic pain symptoms, affective distress, and somatosensory function can help uncover potential underlying central mechanisms of pain after TBI (29, 30). Neuropathic pain symptom severity and the psychosocial impact associated with pain can be evaluated using the Neuropathic Pain Symptom Inventory (NPSI) (31–33) and the Multidimensional Pain Inventory (MPI) (34). The NPSI assesses the presence and severity of common neuropathic pain symptoms, and has been validated to identify neuropathic pain phenotypes across many chronic pain populations (31, 32, 35, 36). However, chronic pain phenotypes based on NPSI scores have not been described in individuals with TBI. Moreover, Section 1 of the MPI measures the impact of pain on individuals' quality of life, including pain severity and interference, affective distress, support from significant others, and general activities. Thereby providing a useful tool to evaluate psychosocial and behavioral aspects associated with neuropathic pain.

Measurements of sensory function can uncover alterations in the somatosensory system (e.g., spinothalamic (STT) and dorsal column medial lemniscal (DCML) tracts), which are commonly present in central neuropathic pain syndromes (35, 37–39). Sensory indicators associated with chronic pain may include negative (e.g., decreased or loss of sensation) and positive somatosensory symptoms (e.g., allodynia or hyperalgesia), indicating functional changes in the nerve tracts responsible for transmitting and processing sensory information (35, 37–39). One approach to investigating positive and negative somatosensory symptoms and signs is quantitative sensory testing (QST) (40). Although previous studies have used QST to evaluate somatosensory function in individuals with pain after TBI (4, 5, 9), these studies have not investigated differences in somatosensory and psychosocial function associated with pain phenotypes. It has been proposed that phenotypes or subgroups based on pain symptoms, somatosensory function (e.g., thermal detection and pain thresholds), or psychological measures (e.g., depression and anxiety) may indicate common underlying pain-associated mechanisms (31, 39, 41–44). For that reason, several studies have attempted to link neuropathic phenotypes based on pain symptoms or signs to treatment outcomes (32, 45, 46). For example, a significant reduction in pain intensity (compared to placebo) after botulinum toxin A administration (42) was observed among only those with either the evoked pain or pressing pain phenotype (44). Thus, the primary purpose of the present study was to evaluate the presence and severity of neuropathic pain symptoms and associated-anxiety symptoms in an effort to identify and profile neuropathic pain phenotypes among individuals with TBI.

We hypothesized that (1) two or more pain phenotypes would be present after TBI and (2) phenotypes would differ in psychological, somatosensory, and psychosocial function. To test our hypotheses, we conducted a two-step cluster analysis to identify pain phenotypes based on the presence and severity of neuropathic pain and anxiety symptoms in individuals with TBI (*N* = 48). Then, we compared the phenotypes on measures of psychological (depression and PTSD), somatosensory (thermal and mechanical detection and pain thresholds), and psychosocial (pain severity, pain interference, support, affective distress, and life control) function. Our approach included: (1) a comprehensive pain evaluation, (2) evaluation of psychological factors, (3) QST, and (4) evaluation of psychosocial factors.

## Materials and methods

### Study participants

Participants were recruited through advertisements posted at the University of Miami Medical Campus and *via* the Health and Human Services/National Institute on Disability, Independent Living, and Rehabilitation Research (HHS/NIDILRR), South Florida TBI Model System center, the Transforming Research and Clinical Knowledge in Traumatic Brain Injury (TRACK-TBI) study center within the University of Miami (UM), and by word of mouth. As per inclusion criteria, all participants were fluent in English, without moderate or severe cognitive impairment as evidenced by the Mini-Mental State Examination, 2^nd^ edition (MMSE-2) (47), had no recent history of alcohol or drug abuse, and had no severe major depression [Beck's Depression Inventory (BDI-II)] (48), or other neurological disease or trauma (e.g., SCI, multiple sclerosis). Participants experienced a closed-head TBI at least 6 months before study participation. They provided medical records with proof of head/brain injury obtained from their medical care provider or insurance company unless they were directly referred from the HHS/NIDILRR, South Florida TBI Model System center, or the TRACK-TBI study center. The severity of TBI was determined based on Glasgow Coma Scale (GCS) (49) when available.

### Study protocol

A two-visit cross-sectional study was conducted at the Clinical Pain Research Laboratory at the University of Miami. We collected demographic, psychological, sensory, psychosocial, and whole-brain neuroimaging information using standardized questionnaires and assessments. The data presented in this article did not include the brain imaging data, as this will be presented separately. The University of Miami Institutional Review Board (IRB) approved the protocol, and all participants provided written informed consent before study assessments.

### Screening instruments

#### Folstein mini-mental state examination, 2nd edition

The MMSE (50) is a brief mental status screen widely used in medical settings. With a potential score of 30, scores below 25 are considered impaired. Subjects with moderate to severe cognitive impairments (i.e., scores of 18 or below) were excluded from the study. The MMSE has demonstrated adequate test-retest reliability (*r* = 0.83–0.89) (50) and has been used to screen for cognitive impairment in those with TBI (Cronbach's alpha = 0.76) (51).

#### Alcohol use disorder identification test

The AUDIT (52) provides an accurate measure of risk for alcohol abuse across gender, age, and cultures. The AUDIT consists of 10 questions about recent alcohol use, alcohol dependence symptoms, and alcohol-related problems. Subjects with scores of 10 or above were not eligible for enrollment. The AUDIT has been used across a variety of populations and has shown strong test-retest reliability (*r* = 0.80–0.85) and average internal consistency metrics (Cronbach's alpha = 0.80) (53).

#### Drug abuse screening test (DAST-10)

The DAST (54) is designed to be used in a variety of settings to provide a simple way to detect drug-related problems. The DAST provides a brief, self-report instrument for population screening, identifying drug problems in clinical settings, and treatment evaluation. Subjects who scored three and above (indicating that moderate to severe substance abuse is present) were not eligible for enrollment. The DAST-10 has shown adequate test-retest reliability (*r* = 0.71) and excellent internal consistency (Cronbach's alpha = 0.86–0.94) (55).

#### Beck depression inventory, 2nd edition

The BDI (56) is a 21-item self-report multiple-choice questionnaire designed to assess depressive symptoms. Participants were required to rate their symptoms over the past 2 weeks from 0 to 3, with increasing scores reflecting greater symptomatology. Subjects who scored above 29 on the BDI (indicating that severe depressive symptoms are present) were excluded as per inclusion/exclusion criteria as severe depression may impair the ability of the participant to cooperate with the study requirements and the completion of all visits/assessments. The BDI has been used in those diagnosed with chronic pain and has shown excellent test-retest reliability and internal consistency (Cronbach's alpha = 0.94) (57).

### Demographic and injury characteristics

Participants provided information regarding demographic and injury characteristics (e.g., age, gender, race/ethnicity, age at TBI, mechanism of TBI, and Glasgow Coma Scale (GCS) scores, if available).

### Psychological assessments

#### Beck's anxiety inventory

The BAI (58) is composed of 21 self-report items used to evaluate the presence and severity of anxiety symptoms within our sample. BAI scores from 0–21 indicated low anxiety, 22–35 indicated moderate anxiety, and a score of 36 and above indicated severe anxiety. The BAI has been used to measure anxiety across a variety of populations, with results indicating adequate test-retest reliability (*r* = 0.75) and internal consistency (Cronbach's alpha = 0.92)(58, 59).

#### Post-traumatic stress disorder checklist-civilian version

The PCL-C is composed of 17 items (60–62) and was used to evaluate the presence and severity of PTSD symptoms. PCL-C scores from 17–29 indicate little to no severity, 28–29 some PTSD symptoms, 30–44 moderate severity of PTSD symptoms, and 45–85 high severity of PTSD symptoms. The PCL-C has been used in previous TBI research (63). Psychometric criteria indicate adequate test-retest reliability (*r* = 0.68) and internal consistency (Cronbach's alpha = 0.94) of the scale across published studies (60, 64).

### Quantitative sensory testing

QST was performed using the method of limits (65) to evaluate mechanical and thermal somatosensory function. Our group previously reported this methodology (66, 67) adapted from the standardized QST protocol of the German Research Network on Neuropathic pain (DFNS) group (68). All QST procedures were performed in a quiet room with a temperature between 21 °C and 23 °C. For each QST modality (i.e., thermal and mechanical), we obtained an average value across trials (3 or 4 depending on QST modality) for each tested site. In addition, since thermal QST thresholds are independent of skin temperature within the range of 27–37 °C (69), we measured the skin surface temperature before starting the procedures to ensure baseline skin temperature was within this range.

#### Tested standard body sites

We conducted the QST on a proximal site (central forehead, V1 dermatome) and a distal site (medial calf, L4 dermatome) relative to the head injury. The forehead was chosen because headache is common after TBI (70). The right medial calf was selected because it is remote from the head injury, and abnormalities detected in this site may indicate central changes.

#### Thermal detection and pain thresholds

For cool and warm detection thresholds (CDT, WDT), the thermode (30x30 mm) from the Thermal Sensory Analyzer (TSA-II Medoc Ltd, Ramat Yishai, Israel) began at 32 °C and decreased or increased at a rate of 1.5 °C/s. Participants were instructed to immediately indicate when they felt the cool or warm sensation. Each procedure consisted of four trials with an inter-stimulus interval of 10-second. Cold and hot pain thresholds (CPT and HPT) consisted of three trials (20-s inter-stimulus interval), during which subjects indicated as soon as they felt pain from the stimulus. If no sensation (detection or pain) was felt, the maximum (50 °C) or minimum (0 °C) temperature of the equipment was recorded. We calculated the difference between the pain threshold and the perception threshold as a measure of sensory function.

#### Mechanical detection and pain thresholds

For vibration detection thresholds (VDT), we used the VSA-3000 probe (1.22-cm^2^ tip). This procedure consisted of three trials (15-s inter-stimulus interval) with probe amplitudes increasing from 0 to 130 μm at 0.5 μm/s and 100 Hz. In addition, pressure pain thresholds (PPT) were measured using a pressure algometer applied perpendicular to the skin area. Gradual pressure was applied at a rate of 30 kPa/s (three trials with 45-s inter-stimulus interval). Participants reported as soon as they felt vibration or pain from corresponding stimuli.

### Pain and psychosocial assessments

Participants completed the Neuropathic Pain Symptom Inventory (NPSI) (33), the Multidimensional Pain Inventory (MPI) (34), and for descriptive purposes, a modified version of the International SCI Basic Pain Dataset - ISCIBPD version 2.0 (71). These questionnaires assessed pain characteristics, neuropathic pain symptom severity, and psychosocial impact associated with pain.

#### Neuropathic pain symptom inventory

The NPSI (33) assesses the severity of 10 descriptors commonly associated with neuropathic pain and two temporal items regarding spontaneous pain and pain attacks. In addition, it includes severity ratings of each descriptor using a 0–10 numerical rating scale (NRS), reflecting the following five dimensions of neuropathic pain: (1) burning superficial spontaneous pain, (2) pressing deep spontaneous pain, (3) paroxysmal pain, (4) evoked pain, and (5) paresthesia/dysesthesia. NPSI descriptors reflecting evoked pain highly correlate with clinician-based quantified measures of allodynia and hyperalgesia in the painful site (72). Total NPSI scores were calculated by adding severity ratings (0 to 10) from each NPSI descriptor (maximum total NPSI score is 100, the two temporal items are not scored). A score of zero was recorded for participants who did not report any neuropathic pain symptoms or chronic pain.

#### Multidimensional pain inventory

The multidimensional pain inventory (MPI) (34) was used to assess the impact of chronic pain. This questionnaire has previously been used and validated in multiple pain populations (73, 74). Section 1 of the MPI provides information regarding pain severity, life interference, support, and life control. Other sections evaluate perceived social support and activities. Sub-scores were calculated by averaging individual scores from specific items rated on a 0–6 scale (34).

#### A modified version of the International SCI Basic Pain Dataset - ISCIBPD version 2.0

Using an interview-based format, participants indicated the anatomical location of their pain on a pain drawing (75). If a participant experienced more than one pain problem (or complaint), and these pain problems were distinguishable from each other, the participant was asked to identify up to three worst pain problems. Then, they were asked to provide the characteristics of the “worst,” “second worst,” and third worst” pain problem, if present. The pain characteristics evaluated included the location, descriptors (e.g., burning), onset date, and treatment course. Additionally, the average pain intensity for each pain problem in the last week was obtained using a 0 to 10 NRS.

### Statistical methods

#### Power analysis and sample size estimation

The present data was aggregated from a larger dataset, including MRI data collection. An *a priori* sensitivity power analysis was conducted for brain imaging measures for the parent MRI study. However, for this substratum of data, we performed an additional sensitivity power analysis using G^*^Power 3 software (76). Findings indicated that a sample size of 38 individuals was large enough to detect differences of interest between two phenotypes using a *t*-test (two independent means) which corresponds to a large effect size of *d* = 1.8 with a statistical power (1–β) of 0.95 (given α = 0.05). This calculation was based on group parameters (mean and standard deviation obtained by pain severity in TBI) reported by previous literature (77).

#### Cluster analysis

Individuals with traumatic brain injury (TBI) may experience neuropathic pain symptoms, which can be associated with affective distress (78). Therefore, it is important to identify pain phenotypes that consider both factors. Since BDI scores were used as a screening criterion to exclude individuals with major depression, we selected BAI scores as indicators of affective distress. Thus, clusters were defined based on the presence and severity of neuropathic pain and anxiety-associated symptoms. For this analysis, total NPSI and BAI scores were used as continuous variables in a two-step cluster analysis to identify multidimensional pain phenotypes after TBI. Total NPSI scores were selected for the cluster analysis because they represent the severity of common symptoms associated with neuropathic pain. Previously, NPSI scores have been used to identify clusters reflecting neuropathic pain phenotypes (31, 32, 35, 36). Our group has used this approach to identify clusters in individuals with pain during the subacute stage after TBI (77) and chronic stage after SCI (31). This approach automatically defines the number of possible clusters by the default criterion (Schwarz's Criterion). Then, the clusters were examined for appropriateness and labeled based on the mean total NPSI and BAI scores. Clusters were validated by comparing them on psychological variables not included in the cluster analysis (e.g., depression and PTSD). The cluster analysis was performed using SPSS software v26.

#### Comparisons between phenotypes regarding somatosensory function

First, arithmetic differences between thermal pain (CPT, HPT) and detection thresholds (CDT, WDT) were calculated and represented as delta (Δ) of CPT-CDT and Delta HPT-WDT. Secondly, independent samples *t*-tests were performed to assess differences in QST CPT-CDT and HPT-WDT on the proximal and distal sites. finally, independent samples t-tests were used to assess differences in mechanical thresholds (i.e., VDT and PPT) on the proximal and distal sites. statistical analyses were performed using SPSS software v26 and GraphPad Prism v9.3.1.

#### Comparisons between phenotypes regarding chronic pain and psychosocial function

Independent samples *t*-tests were used to assess differences in pain characteristics (number of pain problems, location of pain, pain intensity, frequency of pain, onset of pain, and medication), neuropathic pain symptoms (burning spontaneous pain, pressing spontaneous pain, paroxysmal pain, evoked pain and paresthesia/dysesthesia), and psychosocial function (pain severity, life interference, support, affective distress, and life control). Only those who experienced chronic pain were compared on pain and pain-related psychosocial function. Statistical analyses were performed using SPSS**s** v26 and GraphPad Prism v9.3.1.

## Results

### Cluster analysis

**Figure 1:**
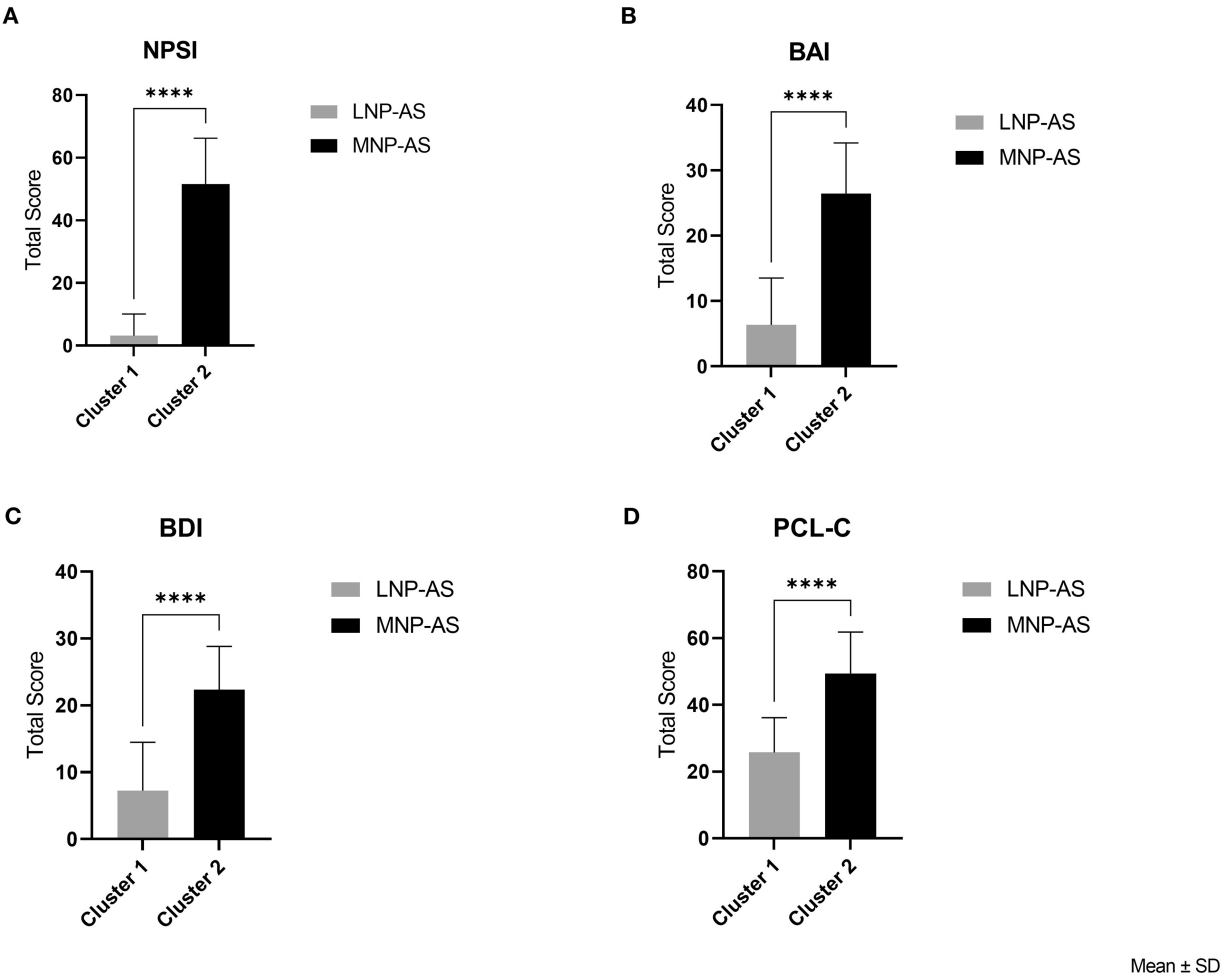
Cluster analysis and validation. Clusters were compared on: **(A)** NPSI (neuropathic pain symptom inventory), **(B)** BAI (Beck Anxiety Inventory), **(C)** BDI (Beck Depression Inventory), and **(D)** PCL-C (Post-traumatic Stress Disorder Checklist-Civilian version) total scores. ****p < 0.0001.

Thirty-eight individuals who had experienced a TBI were included in this study. The cluster analysis revealed two clusters. Cluster 1 (*n* = 27, females = 12, males = 15) had low total NPSI (mean ± SD = 3.2 ± 6.9) and low BAI (mean ± SD = 6.3 ± 7.2) scores, and cluster 2 (*n* = 11, females = 6, males = 5) had moderate total NPSI (mean ± SD = 51.6 ± 14.6) and moderate BAI (mean ± SD = 26.5 ± 7.7, see Figures 1A,B) scores. The two clusters were referred to as low neuropathic pain and anxiety symptoms LNP-AS (Cluster 1) and moderate neuropathic pain and anxiety symptoms MNP-AS (Cluster 2). Cluster 2 had significantly higher BDI scores [*t*_(36)_ = −6.01; *p* = < 0.001], see Figure 1C) and PCL-C scores [*t*_(36)_ = −6.01; *p* = < 0.001], see Figure 1D) than the cluster 1.

### Demographic and injury characteristics of phenotypes

Chi-square and independent-samples Mann-Whitney U tests assessed differences in demographic and injury characteristics (Table 1). The phenotypes were not significantly different. Participants in the LNP-AS who did not experience any neuropathic pain symptoms did not differ from the MNP-AS phenotype regarding demographic and injury characteristics (e.g., time after TBI). The most commonly reported mechanism of the injury in both phenotypes was a motor vehicle accident, which has been previously observed among adolescents and young adults in the civilian TBI population (79). Regarding the time after injury, six participants reported a time after TBI between 6 months and 1 year (4 in the LNP-AS and 2 in the MNP-AS phenotype). The maximum time after TBI in this cohort was 276 months. The severity of the injury was estimated for fourteen TBI participants based on the available Glasgow Coma Scale (GCS), according to the Veterans Affairs/ Department of Defense (VA/DoD) Clinical Practice Guideline for Management Concussion/Mild Traumatic Brain Injury (49). The severity of the injury was not significantly different between phenotypes. Additionally, a sub-analysis indicated that GCS scores were not correlated with total NPSI scores.

**Table 1:** Demographic and injury characteristics.

	**LNP-AS**	**MNP-AS**	**Group difference**	
	**(*n* = 27)**	**(*n* = 11)**	***Test statistic* (*df*)**	***p***
**Gender (** ***n*** **,%)**			χ ^2^(1) = 0.32	0.57
Male	15 (55.6)	5 (45.5)		
Female	12 (44.4)	6 (54.5)		
**Age (years, mean, SD)**	31.6 (9.9)	36.3 (11.2)	*t*_(36)_ = −1.27	0.21
**Race/ethnicity (** ***n*** **, %)**			χ ^2^(3) = 5.98	0.11
African American	3	5		
Asian	2	0		
Hispanic	14	4		
White (Non-Hispanic)	8	2		
**Age at TBI (years, mean, SD)**	25.4 (10.3)	32.3 (10.8)	*U*(1) = 204.50	0.07
**Time after TBI (months, mean, SD)**	74.4 (71.2)*	46.9 (30.7)	*U*(1) = 126.00	0.47
**Severity of TBI (** ***n*** )**			χ ^2^(2) = 2.57	0.28
Mild	3	3		
Moderate	2	0		
Severe	5	1		
**Cause of injury (n)**			χ ^2^(3) = 5.72	0.13
MVA	18	7		
Sport	8	2		
Act of violence	0	2		
Other	1	0		
**MMSE-2**				
Mean (SD)	28.4 (1.8)	27.4 (1.9)	*t*_(36)_ = 1.61	0.12

MVA, motor vehicle accident, MMSE-2, Mini-Mental State Examination – Version 2. *Minimum and maximum time after TBI was 6 to 276 months. **injury severity was not available from 17 LNP-AS and 7 MNP-AS participants.

### Comparisons between phenotypes regarding somatosensory function

**Figure 2:**
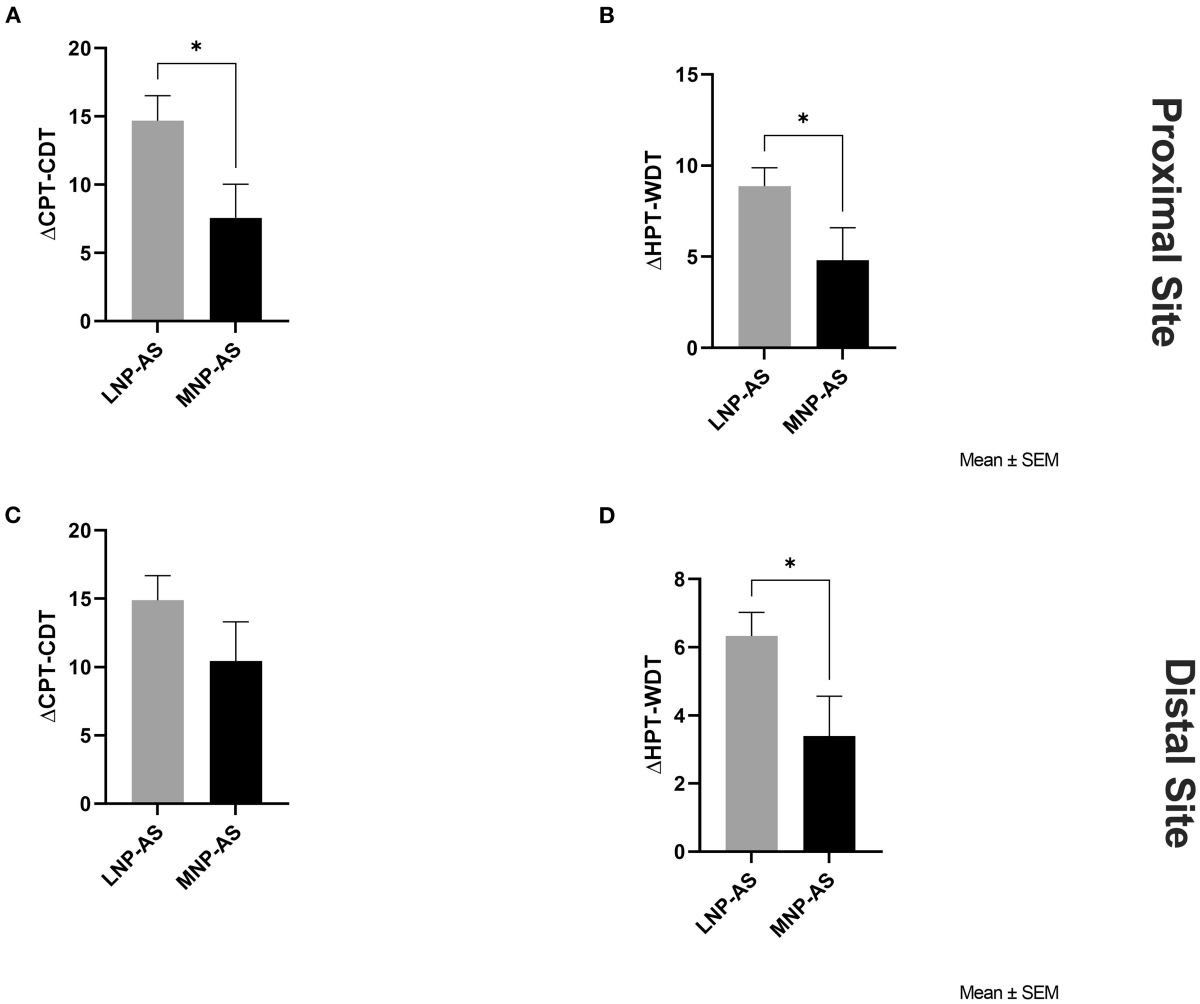
Thermal somatosensory function obtained from QST. Significant group differences were found on the proximal site with respect to: **(A)** ΔCPT-CDT (delta cold pain threshold-cool detection threshold) and **(B)** ΔHPT-WDT (delta hot pain threshold-warm detection threshold); and on the distal site with respect to **(D)** ΔHPT-WDT between the LNP-AS (low or no neuropathic pain-anxiety symptoms) and MNP-AS (moderate neuropathic pain-anxiety symptoms). On the distal site, no group differences were found regarding **(C)** ΔCPT-CDT (delta cold pain threshold-cool detection threshold). *p < 0.05.

**Figure 3:**
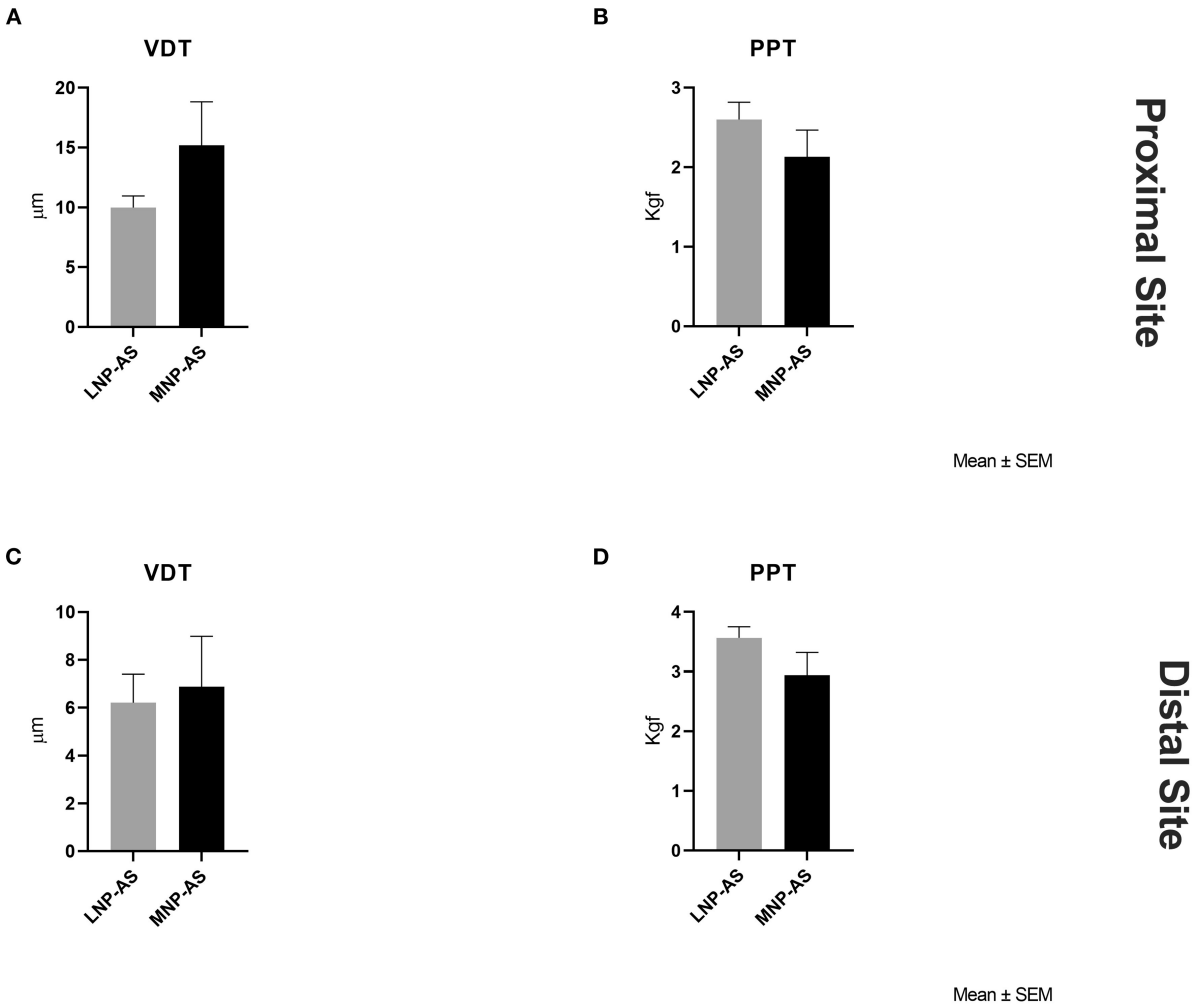
Mechanical somatosensory factors obtained from QST. Group differences were not found with respect to **(A)** VDT (vibration detection threshold) on the proximal site, **(B)** PPT (pressure pain threshold) on the proximal site, **(C)** VDT on the distal site, and **(D)** PPT on the distal site between the LNP-AS (low or no neuropathic pain-anxiety symptoms) and MNP-AS (moderate neuropathic pain-anxiety symptoms).

Differences between phenotypes regarding somatosensory function are displayed in Figure 2. Results from the *t*-tests indicated lower ΔCPT-CDT [*t*_(36)_ = 2.16; *p* = 0.038] and ΔHPT-WDT [*t*_(36)_ = 2.08; *p* = 0.044] at the proximal site in the MNP-AS phenotype compared to the LNP-AS phenotype. No significant differences were found on the distal site with respect to ΔCPT-CDT [*t*_(36)_ = 1.32; *p* = 0.196]. However, there was a significant difference in ΔHPT-WDT [*t*_(36)_ = 2.23; *p* = 0.032] suggesting changes in the central pathways mediating temperature and pain. As shown in Figure 3, no significant differences were found regarding mechanical thresholds (i.e., VDT and PPT) on the proximal site and the distal site. VDT on the proximal site [*t*_(36)_ = 1.89; *p* = 0.067] and distal site [*t*_(36)_ = 0.29; *p* = 0.773]; PPT on the proximal site [*t*_(36)_ = 1.16; *p* = 0.254] and distal site [*t*_(36)_ = 1.64; *p* = 0.110].

#### Comparisons between phenotypes regarding chronic pain and psychosocial function

Of all participants, nineteen reported experiencing chronic pain with moderate intensity (equal to or more than four on a 0–10 NRS. Thus, their pain characteristics were further evaluated in detail and compared between phenotypes (Table 2). Eight of these participants were clustered in the LNP-AS phenotype, and eleven were clustered in the MNP-AS phenotype. Participants clustered in the LNP-AS presented the following total NPSI (mean ± SD: 10.75 ± 9.22), BAI (mean ± SD: 9.88 ± 9.14), BDI (mean ± SD: 8.63 ± 8.52), PCL-C (mean ± SD: 28.50 ± 12.18) scores. Most participants experienced several concomitant pain problems in different body regions. The average pain intensity of the worst pain problem was significantly higher in the MNP-AS phenotype than in the LNP-AS phenotype, and most participants developed their worst pain (or only pain) problem on the date of injury. The most common location for the pain problems in the MNP-AS phenotype was the head, consistent with previous studies in subacute and chronic TBI (4, 77, 80). The head pain was commonly described as throbbing, aching, squeezing, sharp, shooting, penetrating, and stabbing. Eighty-seven percent of the head pain (s) evaluated in the MNP-AS phenotype developed on the date of the TBI. This observation indicates that the acute pain experienced after TBI may transition into chronic pain (81). Additionally, lower extremity and neck/shoulder pain was experienced by ten (90.1%) and eight (72.7%), respectively, of the MNP-AS participants. The most commonly reported medications among participants were NSAIDs (ibuprofen, naproxen, acetaminophen, aspirin, and diclofenac) and opioids (Percocet, oxycodone, and tramadol). Antidepressants such as tricyclic or serotonin and norepinephrine reuptake inhibitors were not reported by the participants.

**Table 2:** Chronic pain characteristics and medication.

	**LNP-AS**	**MNP-AS**
	**(*n* = 8)**	**(*n* = 11)**
**Number of pain problems (** ***n*** **, %)**		
One	1 (12.5)	0 (0)
Two or more	7 (87.5)	11 (100)
**Location of pain problems (** ***n*** **, %)**		
Head	5 (62.5)	11 (100)
Neck/shoulders	6 (75.0)	8 (72.7)
Upper/lower back	3 (37.5)	7 (63.6)
Fontal torso	0 (0)	2 (18.2)
Lower extremity	5 (62.5)	10 (90.1)
Upper extremity	2 (25.0)	2 (18.2)
**Average pain intensity for the worst pain problem (mean, SD)** ^a^	**6.1 (1.8)**	8.5 (1.1)^**^
**Onset of pain after the TBI of the worst pain problem (** ***n*** **, %)**		
On the date of injury	6 (75.0)	10 (91.1)
Months after injury	2 (25.0)	0 (0)
One year after injury	0 (0)	1 (9.09)
**Medication (** ***n*** **, %)**		
NSAIDs	4 (50.0)	6 (54.5)
Opioids	0 (0)	4 (36.4)
Anti-convulsant	0 (0)	2 (18.2)
Anxiolytics	1 (12.5)	2 (18.2)
Cannabis	1 (12.5)	2 (18.2)
None	2 (25.0)	1 (9.1)

a *In the last week*,

** *p < 0.01 (Mann-Whitney Test). Bold indicates significant values. LNP-AS, low neuropathic pain-anxiety symptoms. MNP-AS, moderate neuropathic pain-anxiety symptoms*.

**Figure 4:**
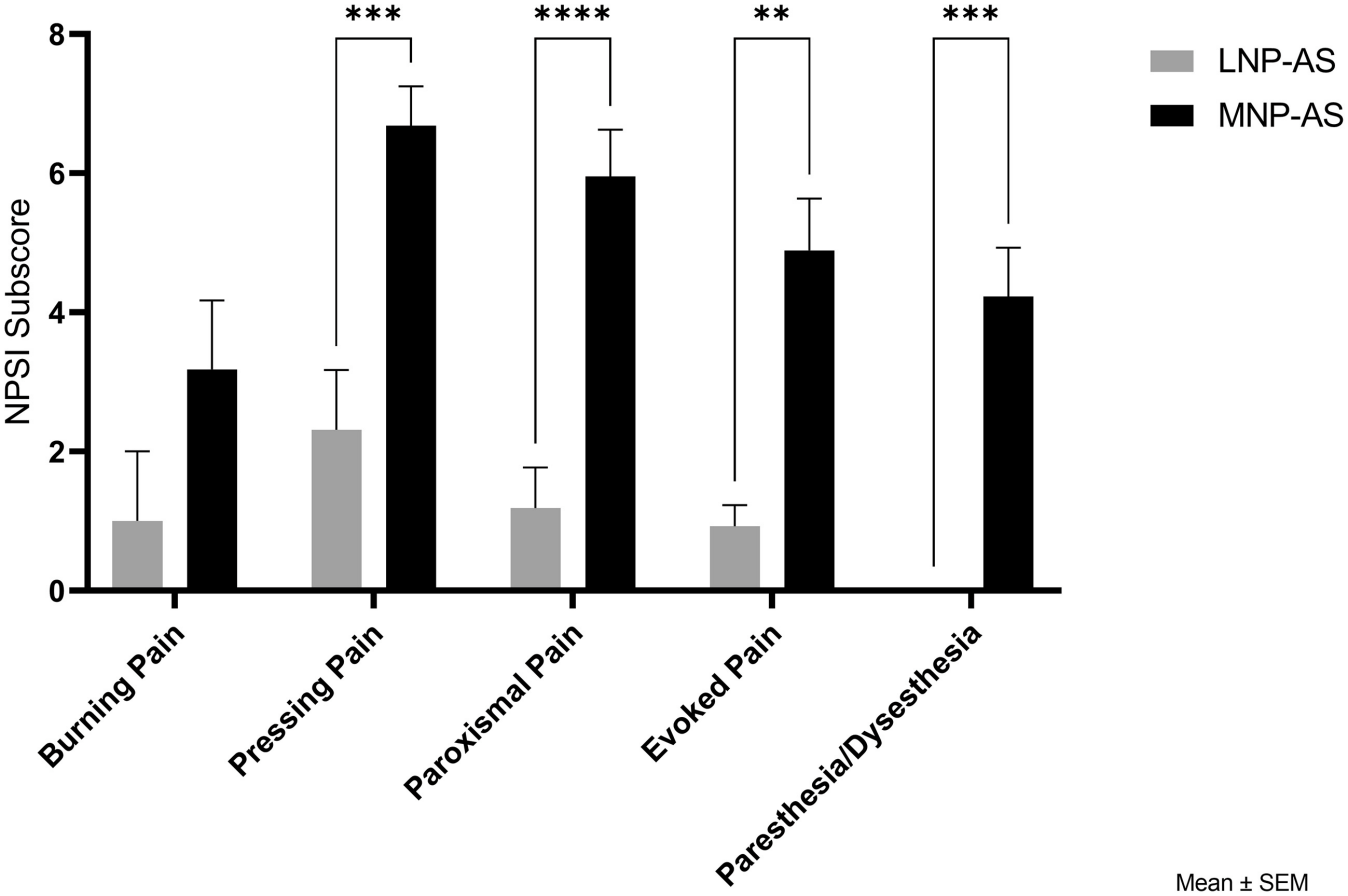
Comparison of phenotypes with respect to NPSI subscores. NPSI, neuropathic pain symptom inventory; LNP-AS, low or no neuropathic pain-anxiety symptoms; MNP-AS, moderate neuropathic pain-anxiety symptoms. **p < 0.01, ***p < 0.001, ****p < 0.0001.

Regarding neuropathic pain symptoms, Table 3 indicates the proportion of patients reporting each neuropathic pain symptom from the NPSI. All participants in the MNP-AS phenotype indicated having pressure and squeezing pain symptoms, and this spontaneous pain was usually present between 8 and 12 h during any given day. Most participants in the MNP-AS phenotype also experienced stabbing and electric shock pain attacks. Pressure evoked pain suggesting the presence of mechanical allodynia was common in both phenotypes. However, only the MNP-AS participants reported cold-evoked pain, pins and needles, and tingling sensations. The mean intensity of each neuropathic pain sub-score (scores 0 to 10 on a numerical scale) representing different dimensions of neuropathic pain is shown in Figure 4. Pressing, paroxysmal and evoked pain, and paresthesia/dysesthesia neuropathic pain sub-scores were significantly higher in the MNP-AS phenotype. Paresthesia and dysesthesia were experienced by 82% of the individuals in the MNP-AS phenotype. Lastly, burning pain was experienced by 55% of the participants.

**Table 3:** Neuropathic pain characteristics.

	**LNP-AS**	**MNP-AS**
	**(*n* = 8)**	**(*n* = 11)**
**Symptoms (** ***n*** **,%)**		
Burning spontaneous pain	1 (12.5)	6 (54.5)
Squeezing spontaneous pain	2 (25)	10 (90.9)
Pressure spontaneous pain	5 (62.5)	11 (100)
Electric shocks	1 (12.5)	8 (72.7)
Stabbing	3 (37.5)	10 (90.9)
Brush-evoked pain	0 (0)	5 (45.5)
Pressure-evoked pain	5 (62.5)	11 (100)
Cold-evoked pain	0 (0)	6 (54.5)
Pins and needles	0 (0)	9 (81.8)
Tingling	0 (0)	9 (81.8)
**Frequency of spontaneous pain during the past 24 h (** ***n*** **, %)**		
Permanently	2 (25)	2 (18.2)
Between 8 and 12 h	1 (12.5)	5 (45.5)
Between 4 and 7 h	1 (12.5)	3 (27.3)
Between 1 and 3 h	2 (25)	0 (0)
Less than 1 h	2 (25)	1 (9.1)
**Number of pain attacks during the past 24 h (** ***n*** **, %)**		
More than 20	0	0
Between 11 and 20	0	1
Between 6 and 10	0	3
Between 1 and 5	4	7
No pain attacks	4	0

*LNP-AS, low neuropathic pain-anxiety symptoms. MNP-AS, moderate neuropathic pain-anxiety symptoms*.

**Figure 5:**
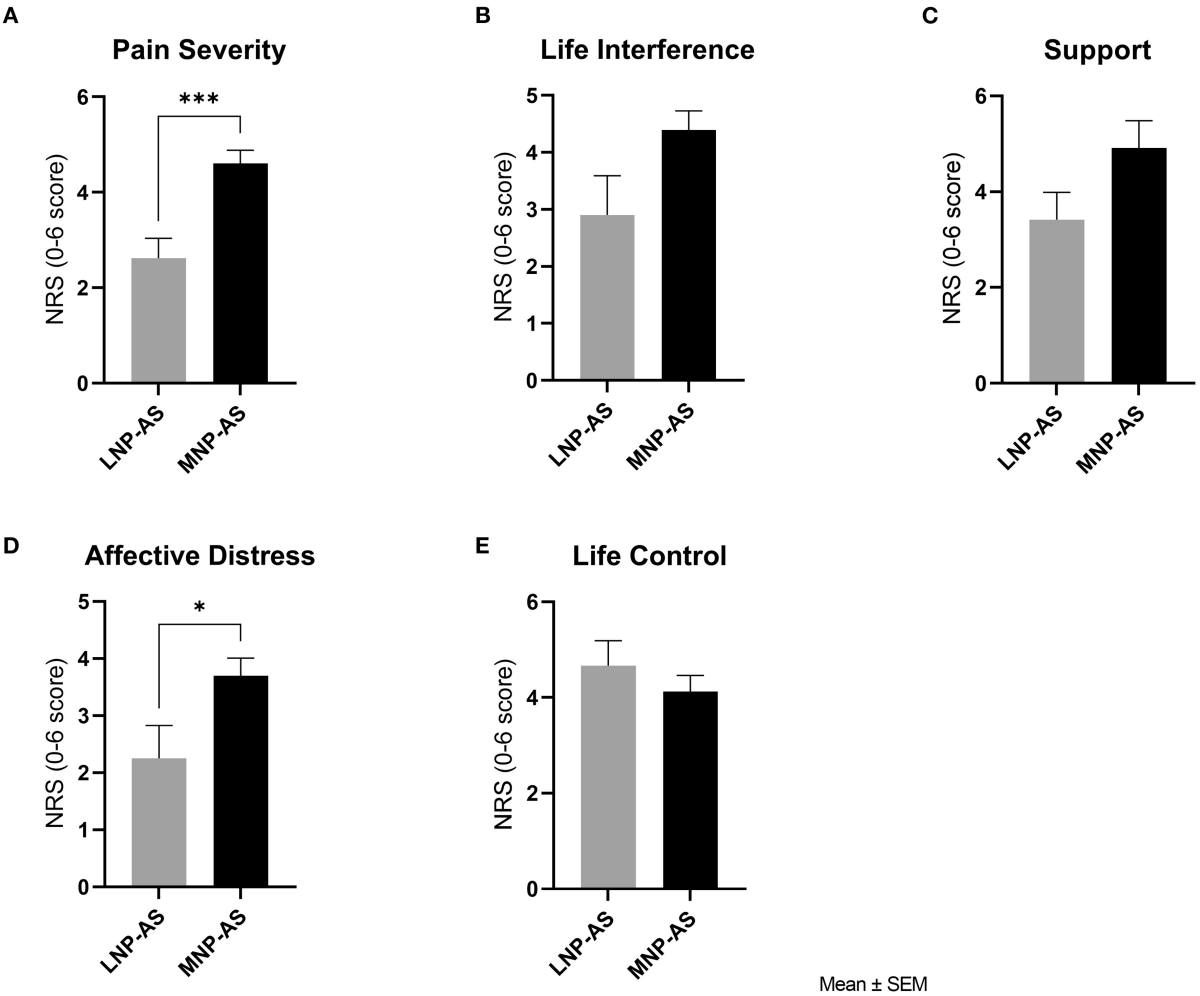
Psychosocial function. Comparison of phenotypes with respect to the Multidimensional Pain Inventory subscales: **(A)** pain severity, **(B)** life interference, **(C)** support, **(D)** affective distress and **(E)** life control. LNP-AS, low or no neuropathic pain-anxiety symptoms. MNP-AS, moderate neuropathic pain-anxiety symptoms. *p < 0.05, ***p < 0.001.

Differences between phenotypes regarding psychosocial function are displayed in Figure 5. Results from the *t*-tests indicated higher pain severity [*t*_(17)_ = 4.14; *p* < 0.001] and affective distress [*t*_(17)_ = 2.38; *p* = 0.02] in the MNP-AS phenotype compared to the LNP-AS phenotype. No significant differences were found regarding life interference [*t*_(17)_ = 2.10; *p* = 0.051], support [*t*_(17)_ = 1.79; *p* = 0.091] and life control [*t*_(17)_ = 0.91; *p* = 0.373].

## Discussion

This study provides a comprehensive description of the characteristics of chronic pain experienced after TBI by evaluating pain symptoms, psychological and sensory function, and psychosocial factors in greater detail than in previous studies. Although the relationship between these factors has been discussed to some extent in heterogeneous pain populations (31, 42, 82), it has not been described in detail in TBI. It is also likely that PTSD, depression, anxiety, fatigue, and sleep disorders are common comorbidities associated with TBI (9, 20–26) and can be etiologically linked to the development of chronic pain (20, 27, 28). Our work builds on this rationale while avoiding any potential confound of severe mental illness or cognitive dysfunction in pain measures. Congruent with these observations, our results show that neuropathic pain symptoms and associated psychological and sensory dysfunction are common in this population. Consistent with our hypothesis, the results confirm the presence of two multidimensional pain phenotypes (based on the severity of neuropathic pain and anxiety symptoms) in individuals following TBI. The phenotypes were characterized by moderate neuropathic pain and anxiety symptoms (MNP-AS) and low or no neuropathic pain and anxiety symptoms (LNP-AS). Furthermore, the MNP-AS exhibited: (1) higher levels of depression and PTSD symptoms, (2) impaired thermal sensory function, and (3) higher levels of pain severity and affective distress compared to the LNP-AS phenotype. These results show that greater neuropathic pain symptom severity after TBI is associated with increased psychological distress and dysfunction in central pathways mediating temperature and pain perception. Although non-significant, we also observed diminished vibration detection thresholds and increased pain detection thresholds in the MNP-AS group, possibly suggesting early dysfunction within central pathways mediating vibration and pressure pain. Although the resulting relationship between neuropathic pain and psychological and sensory function could be expected, our multimodal pain evaluation, including the assessment of neuropathic pain symptom severity, psychosocial impact, affective distress, and somatosensory function can help define specific TBI pain phenotypes to uncover potential underlying central mechanisms of pain after TBI (29, 30). Such an analysis has not been conducted in this population in the chronic TBI stage.

Since chronic pain of different origins is consistently associated with psychological distress and decreased psychosocial function, these factors can also affect a patient's response to treatment. Therefore, multidimensional phenotyping is a promising stratification tool and may have implications for mechanism-based understanding and management approaches to individualized therapy. To date, there is limited research regarding multidimensional pain phenotypes and their characteristics using neuropathic pain symptoms, psychological and sensory function, and psychosocial factors. Particularly following TBI. The MNP-AS cluster identified in this study, presented higher depression and PTSD scores, which supports the association between the severity of neuropathic pain symptoms and psychological distress (82–84). Neuropathic pain associated with TBI has been shown to present a higher incidence of PTSD symptoms, and importantly, the severity of PTSD seems to mediate depressive symptoms (20). Overall, the presence of neuropathic pain in TBI seems to reduce general psychological well-being triggering depressive and PTSD symptoms substantially affecting the quality of life of this clinical population.

In addition to psychological factors, neuropathic pain is associated with the pathophysiological breakdown of somatosensory functions. Studies have found that pain which developed within weeks to months after moderate to severe TBI had sensory characteristics consistent with neuropathic pain (e.g., loss of thermal sensation and evoked pain) (4, 5, 10). Additionally, both basic and clinical research suggests that chronic neuropathic pain after TBI may be associated with several underlying mechanisms, including damage to the spinothalamic and trigeminal systems (4), impaired noradrenergic and serotonergic descending inhibition (6, 85), sensitization (9, 86–88), neuroinflammation (89), and axonal degeneration (90). Also, previous studies on SCI have shown that greater neuropathic pain severity is associated with greater sensitivity to thermal nociceptive stimuli (31, 66, 67). Our data showed that the clusters differed in thermal sensory function at the proximal and distal body sites. Specifically, the MNP-AS exhibited altered thermal sensory function measured by the difference (delta) between pain and detection thresholds, which may indicate impairment of the central pathways mediating temperature and pain *via* the trigeminal and anterolateral sensory systems. Research findings in other neuropathic pain populations have shown that spinothalamic tract (STT) dysfunction (31, 67) and trigeminal system dysfunction (91–93) are associated with the presence and severity of neuropathic pain. Altered thermal sensory function in the MNP-AS cluster may result from central sensitization caused by changes in the expression and function of transient receptor potentials or voltage-gated sodium (Na_v_) channels (94) in Aδ and C fibers.

Regarding mechanical sensory function, the MNP-AS phenotype exhibited higher, though non-significant, VDT and lower PPT values at both the proximal and distal body sites, which has also been observed in other neuropathic pain populations, including SCI (67, 95). Lower vibration sensitivity may indicate sensory dysfunction mediated by the trigeminal sensory system (e.g., dorsal trigeminothalamic tract) and the dorsal-column medial lemniscus (DCML) tract after TBI. Based on clinical and preclinical research, vibratory hyposensitivity may result from neuronal damage, degeneration, or inhibition of Aβ fibers (29, 96), which can cause spontaneous activity in uninjured C fibers (97). Further evaluation of the structural integrity using diffusion magnetic resonance imaging (dMRI) of the trigeminal system and DCML tract could support these results and should be examined in future studies. Conversely, lower pressure pain thresholds may indicate dysfunction of the ventral trigeminothalamic tract, spinal trigeminal tract, STT, or ventral posterior nucleus of the thalamus (6). A supraspinal mechanism that would explain this observed QST profile is an imbalance in convergent inputs from mechanosensitive and nociceptive pathways, as proposed in neuropathic pain following SCI (67, 95). Overall, these findings suggest that greater deficits in central pathways mediating temperature, pain, and vibration are associated with the severity of neuropathic pain symptoms and psychological distress in TBI.

Finally, pain severity and affective distress (characterized by a negative mood and high irritability, tension, and anxiety) were significantly higher in the MNP-AS phenotype than in the LNP-AS phenotype. Pain severity and affective distress have been previously correlated with pain catastrophizing in individuals with mild TBI (98). In addition, the life interference score of the MNP-AS phenotype was higher than in those in the LNP-AS phenotype, indicating an increased burden of pain across various areas of life functioning. Previous studies have reported that pain severity is correlated with life interference, suggesting that the more severe that pain is perceived to be, the greater the probability that it will reduce the level of activity (74, 99, 100); both scores have also been correlated with the presence of insomnia after TBI (101). Interestingly, one study that compared people with chronic pain without TBI vs. people with chronic pain after TBI found that non-TBI individuals reported greater life interference than TBI individuals but no difference in the other MPI scales (102). This was attributed to potential cognitive limitations in TBI individuals. In the present study, cognitive function (evaluated using the MMSE-2) was not different between the resulting phenotypes, suggesting that measures from the MPI were indicators of psychosocial impact without a confounding effect of any cognitive impairment. Additionally, although MNP-AS participants seem to experience greater support (not significantly different from LNP-AS phenotype), they also reported lower life control. Previous research corroborates these findings showing that individuals with TBI report lower control over the pain compared to non-TBIs with chronic pain (102). Similarly, pain severity, life interference, affective distress, support, and life control scores of the MNP-AS phenotype were higher than normative scores from non-TBI patients with chronic pain (*n* = 120) (34). Overall, these results suggest that the severity of neuropathic pain symptoms is associated with greater psychosocial impact.

### Pain phenotypes associated with specific neuropathic pain symptoms

The participants in this study experienced a variety of simultaneous pain problems, and therefore one may argue for the presence of a mixed pain in this population. Although recent publications have discussed the concept of mixed pain (103) (i.e., the simultaneous presence of nociceptive, neuropathic and nociplastic pain where any of these pain components may be the primary pain), a uniform diagnostic strategy regarding mixed pain is currently unavailable. Our study identified an MNP-AS phenotype characterized by higher NPSI pressing and paroxysmal spontaneous pain, evoked pain (e.g., allodynia/hyperalgesia), and paresthesia/dysesthesia compared to the LNP-AS phenotype. Burning pain sub-scores were lower in both phenotypes compared to the other neuropathic pain dimensions. When comparing burning sub-scores in our sample with those of heterogenous pain populations (31, 33, 35, 77, 104, 105), our scores seemed lower. Burning as a symptom of neuropathic pain appears to be more common and severe in people with SCI (31, 104, 106), diabetic peripheral neuropathy, and HIV neuropathy (33, 35) but less common/severe in subacute TBI (77). This may suggest that TBI-related phenotypes represent a less commonly studied presentation of pain than other neuropathic pain conditions. In contrast, pressing pain and paroxysmal pain scores were higher in the MNP-AS phenotype when compared to other subacute TBI studies (77), diabetic peripheral neuropathy (33, 35), HIV neuropathy (33, 35), SCI (31, 104), central poststroke pain (35, 105), and post-traumatic peripheral pain (35). Higher pressing pain and paroxysmal pain severity seem to be more common in the TBI population than in other neuropathic pain populations, indicating that TBI-related pain may depend on partly different mechanisms. Regarding evoked pain, brush-evoked and cold-evoked pain symptoms were only experienced by the moderate neuropathic pain-anxiety symptoms phenotype. Evoked pain sub-scores (pain provoked or increased by brushing, pressure, and cold) in this cluster were similar to scores reported in other neuropathic pain populations (35) and subacute TBI (77) but higher than in SCI (31, 104). Additionally, paresthesia/dysesthesia was present only in the MNP-AS phenotype, and these scores were similar to previous scores reported in SCI (31, 104), central post-stroke pain, and post-traumatic peripheral pain (35). Both similarities and differences in neuropathic pain phenotypes have been found across multiple chronic pain conditions (e.g., polyneuropathy, central pain, fibromyalgia, diabetic peripheral neuropathy, and SCI) (29, 31, 35, 107–109). If comparable neuropathic pain phenotypes can be found among diverse chronic pain populations, a more complete mechanistic account may lead to significant treatment implications.

### Limitations

The present study has some limitations. First, many TBI participants could not provide documentation regarding their injury severity or GCS because they did not have access to their medical records, or no GCS was obtained after their injury. Also, participants who did not report experiencing any chronic pain problems (pain experienced for more than 3 months) with a moderate to severe intensity score (equal to or more than four on a 0–10 NRS) were automatically clustered in the LNP-AS by the clustering algorithm. These participants may have experienced non-neuropathic mild chronic pain (less than 4 on a 0–10 NRS) or acute pain (lasting <3 months and not necessarily associated with the TBI). These pains were not assessed in detail in this study, and it is unknown if this has influenced our results. In addition, the relatively small sample size of the present study and the fact that our sample did not include participants with moderate to severe cognitive impairment or recent history of alcohol or drug abuse may have affected the generalizability of our results. Therefore, future studies with larger sample sizes, should be conducted to further expand and validate the presented phenotypes.

## Conclusion

In conclusion, the results of the present study support the notion that neuropathic pain can develop in chronic TBI. Emerging research suggests that treatment outcomes can be related to specific pain phenotypes. Thus, phenotypes may reflect distinct pathophysiological mechanisms that could be specifically targeted in larger clinical trials. Our preliminary findings, including pain phenotypes following TBI need replication and validation in more extensive studies. Nevertheless, this study expands the current knowledge on sensory and psychological function and psychosocial factors associated with pain at the chronic TBI stage. The multidimensional pain phenotypes described in this study may lead to further research aimed at uncovering tailored treatment strategies. Therefore, future studies in this population should (1) evaluate treatment outcomes based on clinically relevant pain phenotypes, and (2) investigate associations between functional and structural brain alterations and pain phenotypes among individuals with TBI who suffer from chronic pain.

## Data availability statement

The raw data supporting the conclusions of this article will be made available by the authors, without undue reservation.

## Ethics statement

The studies involving human participants were reviewed and approved by University of Miami Institutional Review Board (IRB). The patients/participants provided their written informed consent to participate in this study.

## Author contributions

EW-N and VG designed the study. LF and LR performed the assessments and data entry. LR, EW-N, RV, and EF analyzed the data. EW-N, LR, and NC prepared the manuscript draft. All authors substantially contributed to the interpretation of data and manuscript revision.

## Funding

This work was supported by the US Department of Defense (MR141214 Grant).

## Conflict of interest

The authors declare that the research was conducted in the absence of any commercial or financial relationships that could be construed as a potential conflict of interest.

## Publisher's note

All claims expressed in this article are solely those of the authors and do not necessarily represent those of their affiliated organizations, or those of the publisher, the editors and the reviewers. Any product that may be evaluated in this article, or claim that may be made by its manufacturer, is not guaranteed or endorsed by the publisher.
